# The Needs and Experiences of Post-Treatment Adolescent and Young Adult Cancer Survivors

**DOI:** 10.3390/jcm9051444

**Published:** 2020-05-13

**Authors:** Jennifer M Jones, Margaret Fitch, Jared Bongard, Manjula Maganti, Abha Gupta, Norma D’Agostino, Chana Korenblum

**Affiliations:** 1 Cancer Rehabilitation and Survivorship, Princess Margaret Cancer Centre, Toronto, ON M5G 2C4, Canada; bongardj@mcmaster.ca; 2 Bloomberg Faculty of Nursing, University of Toronto, Toronto, ON M5T 1P8, Canada; marg.i.fitch@gmail.com; 3 Department of Biostatistics, Princess Margaret Cancer Centre, Toronto, ON M5G 2M9, Canada; Manjula.Maganti@uhn.ca; 4 Division of Hematology/Oncology, SickKids Hospital Toronto, Ontario, ON M5G 1X8, Canada; abha.gupta@sickkids.ca; 5 Adolescent & Young Adult Program, Princess Margaret Cancer Centre, Toronto, ON M5G 2M9, Canada; 6 Department of Supportive Care, Princess Margaret Cancer Centre, Toronto, ON M5G 2M9, Canadachana.korenblum@sickkids.ca (C.K.); 7 Division of Adolescent Medicine, Department of Paediatrics, SickKids Hospital, Toronto, ON M5G 1X8, Canada

**Keywords:** cancer survivorship, adolescent and young adult, care transitions, follow-up care

## Abstract

(1) Background: Adolescents and young adults (AYAs) who have been diagnosed with and treated for cancer have unique healthcare needs, but more research is needed to inform developmentally targeted cancer care for this population. The purpose of the current analyses was to describe the physical and psychosocial concerns and experiences of AYA cancer survivors during the post-treatment phase. (2) Methods: A national survey was conducted by the Canadian Partnership Against Cancer to evaluate the experiences and unmet needs of cancer survivors (≥18 years) within the first 5 years following cancer treatment. The current analyses were conducted on the AYA survivor population (18–34 years). (3) Results: A total of 575 surveys were completed by AYAs. Of these, 61% were female, 51% were married/partnered, and 52% were 1–3 years post treatment. Approximately three-quarters report their physical or emotional health as good/very good. Overall, 88% reported at least one physical concern [mean of 3.98+2.11 physical concerns (range 0–9)], 90% reported at least one emotional concern [mean of 3.77+1.75 emotional concern (range 0–6)], and 79% reported at least one practical challenge [mean of 2.39+1.28 practical concerns (range 0–5)]. The most common concerns were anxiety/worry about cancer returning (83%), fatigue/tiredness (78%), and depression/loss of interest in daily activities (66%). On average, 43% of those reporting a concern sought help. Common reasons for not seeking help included not wanting to ask, being told that it was normal to feel the way they did, or embarrassment. Of those who did seek help, 37% encountered difficulty obtaining assistance. (4) Conclusions: These results suggest that post-treatment AYA cancer survivors have a high rate and number of physical, psychosocial, and practical concerns and are often not seeking or receiving help to address these. Proactive approaches to characterizing and eliminating barriers to obtaining appropriate care are needed.

## 1. Introduction

Cancer continues to be the leading disease-related cause of death in adolescents and young adults (AYA; defined as ages 15-39) [1,2,3]. However, due to advances in treatments, the overall 5-year survival rate is now over 80% in North America [4]. This positive trend has led to advocacy for the development of specialized follow-up care and survivorship programs for this unique population [1,5,6,7,8].

Given their dynamic developmental stage, the diagnosis and treatment of cancer during early adulthood can result in medical and psychosocial needs that are especially complex [8,9,10,11] and negatively impact and delay the achievement of important life milestones including continuing education, job attainment and progression, and establishing partnerships and families [12,13]. Further, almost all types of cancer treatment can result in physical and psychosocial side effects that may persist after treatment ends and pose life-long risk for the development of late adverse effects [14,15,16,17] and can impair overall quality of life [18] and present AYA cancer survivors with significant and unique challenges in order to restore and sustain their health and overall wellbeing [14,19].

To date, most data on the long-term adverse effects and risks in AYA cancer survivors have been derived from studies of childhood cancer survivors [20,21]. Documented medical late effects in AYA cancer survivors include second primary cancers and endocrine disorders such as infertility, cardiovascular, pulmonary, and neurological complications [22]. Clinically relevant cognitive and psychological late effects are also prevalent in this population, including post-traumatic stress, depression, and anxiety [23]. Both physical and psychological effects in AYA cancer survivors may vary based on cancer site, treatments received, and age at diagnosis and treatment [22]. 

Despite calls to develop effective models to maximize post-treatment care for this population, many AYA survivorship issues continue to be poorly understood and managed [1]. Further, adult cancer centers, where almost all AYA survivors >18 years receive treatment and follow-up care, have limited infrastructure or expertise to address the specific care needs of this patient population [19]. Thus, it is not surprising that AYAs have high levels of unmet needs during and after treatment for cancer [1,24,25,26,27], which can negatively affect quality of life [28,29,30]. 

While it is clear that AYAs likely experience different biopsychosocial outcomes compared to childhood or older adult survivors, research on this unique population remains underrepresented in the literature [1]. There remains a gap in the evidence base to support the care of AYAs survivors, particularly in the follow-up survivorship period when unmet needs are often at their highest [27]. The objective of the current paper is to describe the physical, emotional, and practical concerns in a large sample of Canadian AYA cancer survivors (ages 18–34 years) within the first 5 years post-treatment. The findings help contribute to a better understanding of AYA cancer survivors’ needs; identification of priority issues; and to inform the development of tailored guidelines, services, and resources for this underserved population.

## 2. Methods

A cross-sectional descriptive analysis was conducted using responses from the *Experience of Cancer Patients in Transition Study* (“Transitions Study”), a large national survey administered across ten Canadian provinces conducted by the Canadian Partnership Against Cancer (CPAC). The objective of the Transitions Study survey was to identify the needs of cancer survivors 1–5 years post-treatment and to identify risk factors associated with unmet needs. The methods including survey development, sample selection, and survey dissemination have been described in detail in Fitch et al. 2018 [31]. In brief, the survey was developed based on a literature review and consultations with cancer survivors, clinicians, and system leaders resulting in a conceptual framework. This information was used to develop the survey items. The survey then underwent pilot testing with cancer survivors (*n* = 15) through cognitive interviews and additional survivors (*n* = 96) for performance testing [31]. Both steps included AYA cancer survivors to ensure that the content was relevant. The final version of the survey contained closed and open-ended items and included questions regarding demographic information, health and well-being, cancer history, provision of follow-up cancer care, overall experiences with follow-up, access to follow-up cancer care plans/medical records, health insurance, Internet use, and final comments. The survey was also translated into French. A copy of the survey is available on the CPAC System Performance site http://www.systemperformance.ca/transition-study/.

Eligible patients were identified through provincial cancer registries and included anyone ≥18 years who had completed treatment for cancer. Participants could complete the survey on paper or on-line and in French or English. A total of 40,790 surveys were distributed across Canada in 2016, and 13,258 surveys were returned completed (response rate 33%). Eighty-two percent completed the survey on paper. The study underwent REB and privacy review through the respective cancer registries within all ten provinces.

The data from the Transitions Study were made available through CPAC and accessed in June 2019. A total of 13,319 surveys were completed and data from *n* = 575 respondents ages 18–34 were extracted for the current analyses.

All procedures were in accordance with the ethical standards of the institutional and national committee on human experimentation and with the 1964 Helsinki Declaration. For the current paper, there was no interaction with patients directly as we acquired data from CPAC. The CPAC Transitions study underwent REB and privacy review through the respective cancer registries within all ten provinces.

### Analysis

The analysis focuses on the survey questions regarding physical, emotional, and practical concerns as well as follow-up care provisions and access of support services. Potential concerns were listed within each domain, and respondents were asked to rate how much of a concern each item was (*not a concern, small, moderate, big*). Any endorsed concern (big, moderate or small) was considered a concern and those rated as “big” were defined as severe. If a concern was endorsed, respondents were then asked if they sought help for this concern (*yes/no*). If they did seek help, they were then asked how difficult it was to get help for this concern (*Very easy, easy, hard, very hard, and did not get help*). Difficulty getting help included responses “hard”, “very hard”, and “did not get help”. Descriptive statistics (frequency and proportions) were calculated for demographic and clinical variables and for all relevant survey items including all physical, emotional, and practical concerns; help seeking and difficulty obtaining help for each concern; as well as questions related to access to information, support, and counselling. The mean number of needs (+SD) were calculated for each domain. Data were analyzed using SPSS 21.0 (IBM Corp., Armonk, NY, USA).

## 3. Results

The demographic and clinical characteristics of the AYA respondents are presented in Table 1. The sample included 61% females, of which approximately half were either married or partnered, 60% had post-secondary education, and 65% were currently employed (full or part-time). The most common cancers reported by the AYA sample were hematological (24%), thyroid (16%), and testicular (13.5%). 

The majority of respondents rated their overall physical (76%) and emotional health (71%) as “good” or “very good”. Just under two-thirds (61%) reported that they found it easy (either *very easy* or *easy*) to cope with the challenges of day-to-day life. However, less than half (44%) found it easy to share their worries or concerns with others. 

Considering all services respondents received after treatment completion related to physical, emotional, and practical needs, the large majority (83%) rated the service as good (rated as “very good” or “good”). In terms of access to services, most reported getting the right services (82%), getting the services when they were needed (77%), and receiving follow up care that was personalized to their needs (77%) as good. Finally, just over two-thirds (68%) of respondents said that it was “very easy” or “easy” to ask their doctors questions about their concerns related to follow-up cancer care. 

### 3.1. Physical Concerns

Physical concerns are presented in Table 2. The large majority of respondents (88%) experienced at least one physical concern, and 63% experienced ≥3. The mean number of physical concerns reported was 3.98 ± 2.11 (range 0–9). The most frequently reported physical concerns were fatigue/tiredness (78%), hormonal/menopause or fertility (50%), and changes to concentration or memory (49%). In individuals who identified a concern, the most common physical concerns rated as “big” included hormonal/menopause or fertility (50%), fatigue/tiredness (43%), changes to memory or concentration (36%), and changes in sexual activity or function (35%). 

In terms of seeking help, individuals most frequently sought help for changes in hormonal/menopause or fertility (66%) and chronic pain (66%). However, less than half of respondents sought help for changes in sexual activity/function (38%), changes to concentration or memory (41%), and fatigue/tiredness (47%). A substantial minority of respondents reported difficulty obtaining help for most symptoms (range 22%–47%) with the highest rates of difficulty for changes to their concentration and memory (47%) and sexual activity or function (40%). 

The most common reason individuals with physical concerns did not report their concerns was because someone had told them it was something normal to experience, and they did not think anything could be done about it (30%). 

### 3.2. Emotional Changes

Emotional concerns are presented in Table 3. Ninety percent of survey respondents reported a minimum of one emotional concern, and 64% expressed ≥ 3 concerns. The mean number of emotional concerns reported was 3.77±1.75 (range 0–6). The most commonly reported emotional concerns were anxiety, stress, or worry about cancer returning (84%); depression, sadness, and loss of interest in daily activities (66%); and a change in body image (64%). In individuals who identified a concern, the most common emotional concerns rated as “big” were anxiety, stress, and worry about cancer returning (40%); changes to body image (37%); depression, sadness, and loss of interest in everyday things (33%); and changes to sexual intimacy (33%). 

Respondents most often reported seeking help for depression and sadness (43%) along with anxiety or worry about cancer returning (40%). Fewer sought help for changes in relationships with friends or colleagues (19%), sexual intimacy (25%), or body image (25%). Of those who actively sought help, respondents had the most difficulty finding help for changes in sexual intimacy (46%) and managing changes in relationships with friends or coworkers (42%) and relationships with family and partners (38%). 

The most common reasons for not seeking help for emotional concerns were because they did not want to ask (30%), embarrassment (15%), and that because someone had told them it was something normal to experience, they did not think anything could be done about it (14%).

### 3.3. Practical Challenges

Practical concerns are presented in Table 4. Seventy-eight percent of respondents reported at least one practical challenge and 33% experienced ≥ 3. Respondents reported an average of 2.39±1.28 practical concerns (range 0–5). The most commonly reported practical concerns were returning to work or school (62%), difficulty getting health or life insurance (41%), and getting to and from appointments (37%). The most common practical concerns rated as “big” were returning to work or school (48%) and getting life or health insurance (39%). 

The majority of respondents did not seek help for their practical concerns. The highest frequency of help seeking was for paying health care bills (43%) and returning to work or school (39%). Respondents had the most difficulty getting help for health or life insurance (63%). 

Not wanting to ask for help was the most common reason for not seeking help (24%) along with not knowing services were available (23%) and not knowing where to go to access services (18%). 

### 3.4. Provision of and Access to Information, Support, and Counselling

Respondents were asked to rate how much they agreed or disagreed with statements about the information they were provided. Just over three-quarters of respondents reported that they agreed (rated as “strongly agreed” or “somewhat agreed”) that information was available when they needed it (78%) and was useful to them (78%). While most respondents agreed that they were given information about side effects of treatments (79%), fewer agreed that they were given information about signs of cancer returning (56%) or were given information about the community resources available to them (43%). Most respondents agreed that they received the most useful information about their physical concerns (70%) and less about their emotional (50%) and practical (52%) concerns. 

The minority of respondents had accessed support or counselling services after the completion of treatment. The most common services reported were one-on-one counselling with a professional (28%), on-line peer support through social media (12%), and face-to-face peer support group (8%). Most often respondents were unaware of these resources. Many of the respondents reported using social media or accessing websites for cancer-related information and support. The most common activities included reading what others posted (61%), watching videos (55%), and 55% reported that they actively shared information and links to articles with others on social media.

## 4. Discussion

This study explores the physical, emotional, and practical concerns of young adults (18–34 years) who have been diagnosed and treated for cancer and describes their experiences of care. To our knowledge, this is the largest cohort of post-treatment AYA cancer survivors surveyed about their heath in survivorship. Encouragingly, three-quarters of respondents rated their overall physical and emotional health as good or very good. However, a significant minority of respondents (40%) reported that they were struggling to cope with the challenges of everyday life, and more than half (56%) found it difficult to share their concerns and worries with others. Respondents also reported a significant number of physical, emotional, and practical concerns and often had difficulty finding help for these concerns. Gaps in information provision, especially regarding emotional and practical concerns were identified. Most respondents were regularly using social media and websites to find information and support after the completion of cancer treatments and were not aware of or accessing formal support or counselling services. 

Most respondents endorsed at least one physical concern, and many respondents did not seek help for their physical concerns because they did not think anything could be done to help them or they were told it was normal. Age matched controlled studies have found that AYA cancer survivors report greater medical and physical conditions and more health-related disability and functional limitations than the non-cancer controls [32,33,34]. As reported in the older adult population of cancer survivors, fatigue was the most prevalent concern reported [31]. A recent systematic review of cancer-related fatigue in AYAs highlighted gaps in research on the prevalence, severity, and impact of fatigue, suggesting that more study is needed in this area specific to AYAs [35]. Interestingly, the research to date has found fatigue to be more severe and more common compared to older cancer patients [35,36,37]. 

Concern regarding sexual function/activity was also very common (41%) in our sample. This finding is supported by the recent AYA HOPE cohort study that found 49% of AYAs reported negative effects on sexual function up to 2 years post-treatment [38]. Unfortunately, the majority of respondents in the current study who reported concerns regarding sexual function did not seek help (only 38% did). While patients and clinicians alike may be uncomfortable discussing these issues, these findings suggest the need for routine systematic screening to assess sexual function in AYA cancer survivors. Recent guidelines to address sexual problems in people with cancer have been developed by both ASCO [39] and CCO [40] and may be a helpful resource for clinicians and could be further adapted for AYA population

The most severe physical concerns reported were hormone and fertility concerns, which were reported by half the sample, and half reported this as a severe concern. This was also the physical concern that individuals most frequently sought help for (66%) and, encouragingly, most were able to find help fairly easily (72%). 

Cognitive changes have not been well documented in this population, as most of the research on neurocognitive problems has focused on survivors of childhood cancers [41] or older adult survivors [42]. In this study, almost half of the respondents reported changes to their concentration and/or memory, which is higher than that reported in the older age group (21%) [31] and is of concern given the potential for negative impacts on vocational functioning and employment and education outcomes [43]. Only 41% of those with cognitive concerns sought help and almost half (47%) experienced difficulty finding help. This highlights the need for the healthcare team to identify and validate these concerns and for the development of accessible interventions to address cognitive problems with AYA cancer survivors, which may include cognitive training and compensatory strategy training [44]. In addition, medical, psychosocial, and AYA teams (where they exist) should help advocate for school and/or work accommodations when needed. 

Emotional concerns were also highly prevalent and often severe. While psychosocial issues are common among all cancer populations, they are inversely associated with age [45,46], and unmet psychological and emotional needs have been reported as high concerns in the AYA population [11,47]. As outlined by Zebrack and Isaacson, while AYAs with cancer experience the normal psychosocial stressors related to this developmental transition period, the diagnosis of cancer presents additional and significant challenges [14,48], and AYAs often do not have the life experience or skills to cope effectively [48]. Further, AYA survivors often receive little preparation for the post-treatment survivorship phase, report that their friends and family often do not understand what they are going through, and have a hard time coming to terms being “neither sick nor healthy” [49]. 

In the current study, AYA respondents reported an average of almost four emotional concerns (out of a possible 6). Anxiety and worry about cancer recurrence and depression and loss in interest in daily activities were most common and at rates much higher than the older adult population [31]. Previous research has reported higher levels of emotional issues in AYA cancer survivors compared to aged match controls [32,34,50]. A substantial minority (~40%) of those with anxiety or depression-related concerns sought help and, encouragingly, most were able to find it. Body image concerns and changes to sexual intimacy were also quite common, a finding supported by previous research [33,51,52,53]. The impact of treatment on physical appearance such as hair loss, scarring, and weight gain can result in body dissatisfaction [54], feelings of shame, and affect their sexual identity and sexual health [52,55]. This comes at a time when individuals may be more aware of their body and beginning new intimate relationships [33,51,52,53]. Unfortunately, very few individuals with body image concerns looked for help (25%). 

The main reasons in this study that respondents with any emotional concern gave not to seek help were embarrassment and not wanting to ask or because they were told it was normal, and they did not think anything could be done about it. Unfortunately, most interventions that address psychological and emotional concerns have been developed and targeted to adults or children and are not specific to or have been evaluated in AYA populations [56]. The lack of age appropriate tailored services and interventions for the AYA population may be one reason they do not try to access services. AYA-focused interventions delivered on platforms that are acceptable and accessible to AYAs are urgently needed. Embarrassment about discussing topics related to body image and/or sexuality is also a common barrier [57] and may prevent AYAs from having an open discussion about their needs and concerns. This is especially true for AYAs who identify as a sexual and/or gender minority [58]. While some AYAs prefer healthcare providers to offer face-to-face opportunities to discuss these issues, many younger patients may prefer practical written information or links to on-line information [57]. Bolte and Zebrack (2008) provide a very practical guide to initiating conversations about sexual issues and addressing sexuality in this population and suggest addressing sexual health issues with AYAs soon after diagnosis in order to normalize these discussions throughout treatment and survivorship and to have a member of the treatment team designated as the “sexual health expert” to ensure systematic and standardized assessments and information provision [51].

Practical concerns were very commonly identified, with the most frequently endorsed concerns relating to return to work or school and getting insurance. These were also the most common big concerns, and most did not seek help for them. Work and education are important factors in the development of a sense of identity and in achieving independence [59]. While most AYAs are able to return to work/school after cancer treatment, they often report the need to adapt and reduce job tasks and hours of work due to side effects of treatment [59,60,61]. Given that much of a person’s wage growth occurs in the first decade of their career, delays or breaks in school and employment as a result of a cancer diagnosis may impact on long-term career opportunities and lifetime earnings and ultimately financial status [48]. Return to work and school programs can be effective to help with re-entry and negotiating appropriate accommodations, which can help with successful reintegration [62]. Programs that support AYAs with their schoolwork and that encourage their peer relationships with classmates can also be helpful. 

Finally, while most respondents reported that information was available when they needed it and was useful to them, information regarding cancer recurrence, emotions, and practical concerns as well as community resources was lacking. Further, few respondents reported accessing support or counselling services after the completion of treatment, and most often respondents were unaware of these resources. 

The transition from active cancer treatment to follow-up care presents a critical opportunity for healthcare providers and systems to match their support to self-identified needs and goals of AYA. Consensus exists that AYA-specific post-treatment care models are needed to optimize medical and psychosocial outcomes, and international efforts to establish and evaluate innovative models are increasing [13]. Acknowledging that local program development depends on resources, infrastructure, and region-specific assessment of unmet needs [63], questions such as which professionals in which settings are best suited to deliver this care are still to be determined. Data from this study, from which we can infer that a normalizing, validating approach is essential to combat stigma around or reluctance to seek help, will help inform answers.

## 5. Study Limitations

The results of this study need to be interpreted within the context of its acknowledged limitations. The overall response rate for this survey was 33%, and this may introduce response bias. Further, due to confidentiality issues about characteristics of the respondents, there was insufficient detail to allow for weighing of the surveys to ensure they were representative of all Canadians. In addition, while the developers did include AYA individuals during the review stage of the questionnaire design, and they felt the survey addressed the issues that concerned them, the survey was not specifically developed for AYA cancer survivors. The survey was only offered in English and French. Finally, clinical information including disease status, time since diagnosis, and treatments received was self-reported and was not validated. 

## 6. Conclusions

These results contribute to the growing data on the unmet needs of post-treatment AYA cancer survivors and support the growing call for the development of tailored and accessible interventions and programs that address their specific medical, psychosocial, practical, and informational needs. Further, given the high number of unmet concerns and reported difficulty finding help for these concerns, our findings suggest the need for integrated systematic screening during acute and follow-up care in order to proactively identify issues and respond with developmentally appropriate support and referrals. Encouragingly, advancements have been made in the development of AYA-specific clinical practice guidelines, which were first published in 2012 and were recently updated [64]. These recommend AYA patients be managed by a multidisciplinary team of providers who have expertise in cancer treatment and management of specific AYA developmental issues and that all AYA patients undergo a comprehensive assessment after the diagnosis of cancer. Further, AYA programs built as extensions of adult oncology or pediatric oncology programs have also started to emerge [19,65,66], and descriptive data from this and other studies can be helpful in development and refinement of these efforts.

## Figures and Tables

**Table 1:** Description of adolescents and young adults (AYAs) sample (*n* = 575).

Characteristic	*N*	%
**Sex**		
Male	221	38.4
Female	349	60.7
Other/Prefer not to answer	5	0.8
**Age**		
18–24	116	20.2
25–29	213	37.0
30–34	246	42.8
**Marital Status**		
Single	261	45.4
Married/Partnered	292	50.8
Separated/divorced	16	2.7
Prefer not to answer	6	1.0
**Education**		
High School or less	220	38.3
Post-secondary degree	285	49.6
University graduate degree	58	10.1
Missing	12	2.1
**Income**		
<$25,000	82	14.3
$25,000 to <$50,000	94	16.3
$50,000 to <$75,000	98	17.0
$75,000 or more	220	38.3
Prefer not to answer	75	13.0
Missing	6	1.0
**Employment**		
Employed (full or part time, on leave)	413	71.8
Not employed (homemaker, student)	110	19.1
Unemployed	36	6.3
Missing	16	2.8
**Place of residence**		
Rural (<10,000 people)	121	21.0
Urban (≥10,000 people)	448	77.9
Missing	6	1.0
**Disease Site (most common)**		
Hematological	139	24.2
Thyroid	92	16.0
Testicular	77	13.4
Breast	49	8.5
Melanoma Skin Cancer	47	8.1
Gynecological	39	6.8
Central Nervous System/Brain	33	5.7
Sarcoma	24	4.2
Gastrointestinal	18	3.1
Other	41	7.0
Missing	20	3.5
**Metastases**		
No metastases	433	75.3
Living with metastases	83	14.4
Unsure/Missing	59	10.2
**Time since treatment**		
< 1 year	63	11.0
1 year to < 3 years	299	52.0
3 years to 5 years	170	29.6
No treatment received	38	6.6
Missing	6	1.0
**Type of Treatment**		
Surgery only	172	29.9
Drug therapy only (chemo/non-chemo)	86	15.0
Radiation therapy only	11	1.9
Combination of therapies	290	50.4
No treatment/active surveillance	10	1.7
Missing	6	1.0
**Comorbidities (4 Most Common)**		
Mental Health Issues	71	11.5
Respiratory Diseases	43	6.9
Cardiovascular	11	1.8
Arthritis/Osteoarthritis	8	1.3
**General Physical Health**		
Very poor/poor	19	3.3
Fair	115	20.0
Good/Very good	440	76.5
Missing	1	0.2
**General Emotional Health**		
Very poor/poor	24	4.2
Fair	142	24.7
Good/Very good	402	69.9
Missing	7	1.2
**Overall quality of life**		
Very poor/poor	13	2.3
Fair	63	11.0
Good/Very good	499	86.8
Missing	0	0

**Table 2:** Physical symptoms prevalent and ease of accessing help in AYA sample.

Physical Symptoms	Number of Respondents Who Answered the Question	Number of Respondents Indicating a Concern about a Physical Symptom (Mild, Moderate, or Big)	Number of Respondents Experiencing a Physical Symptom Whose Concern was ‘Big’	Number of Respondents Experiencing a Physical Symptom Whose Concern was ‘Moderate’	Number of those Experiencing a Physical Symptom Who Sought Help^*^	Number of those who Sought Help for their Concern that Experience Some Level of Difficulty (Hard or very Hard to Find Help/no Help Obtained) ^*^
Fatigue or tiredness	566	444 (78%)	192 (43%)	141 (32%)	207 (47%)	73 (35%)
					*N* = 441	*N* = 207
Hormonal, menopause, or fertility	561	280 (50%)	141 (50%)	74 (26%)	184 (66%)	55 (30%)
					*N* = 279	*N* = 184
Changes to concentration, memory	560	274 (49%)	98 (36%)	89 (32%)	112 (41%)	52 (47%)
					*N* = 274	*N* = 111
Nerve problems (numbness or tingling)	562	244 (43%)	55 (23%)	80 (33%)	146 (60%)	44 (30%)
					*N* = 243	*N* = 146
Changes in sexual activity or function	561	229 (41%)	80 (35%)	68 (30%)	86 (38%)	34 (40%)
					*N* = 229	*N* = 86
Chronic pain or long-term pain	561	209 (37%)	52 (25%)	75 (36%)	137 (66%)	51 (38%)
					*N* = 209	*N* = 136
Gastrointestinal problems (i.e., digestion and/or bowl issues)	559	180 (32%)	55 (31%)	57 (32%)	115 (64%)	25 (22%)
					*N* = 180	*N* = 115
Swelling of arms or legs	560	106 (19%)	28 (26%)	29 (27%)	61 (58%)	17 (28%)
					*N* = 105	*N* = 61
Bladder and/or urinary problems (e.g., incontinence)	560	58 (10%)	15 (26%)	19 (33%)	37 (64%)	13 (35%)
					*N* = 58	*N* = 37

* Note: Those who did not answer relevant questions were excluded from the data set. *N* refers to the denominator used for a concern.

**Table 3:** Emotional concerns prevalent and ease of accessing help in AYA sample.

Emotional Concerns	Number of Respondents Who Answered the Question	Number of Respondents Indicating a Concern about an Emotional Issue (Mild, Moderate, or Big)	Number of Respondents Experiencing an Emotional Issue Whose Concern was ‘Big’	Number of Respondents Experiencing an Emotional Issue whose Concern was ‘Moderate’	Number of those Experiencing an Emotional Issue Who Sought Help^*^	Number of those Who Sought Help for their Concern that Experience some Level of Difficulty (Hard or very Hard to Find Help/no Help Obtained) ^*^
Anxiety, stress, or worry about the cancer returning	560	468 (84%)	185 (40%)	150 (32%)	181 (40%)	54 (30%)
					*N* = 465	*N* = 181
Changes in body image (i.e. confidence in appearance)	568	365 (64%)	136 (37%)	119 (33%)	90 (25%)	28 (31%)
					*N* = 359	*N* = 90
Depression, sadness, and loss of interest in everyday things	547	360 (66%)	119 (33%)	121 (34%)	154 (43%)	53 (35%)
					*N* = 355	*N* = 153
Changes in relationship with family, partners	566	274 (48%)	82 (30%)	94 (34%)	80 (30%)	30 (38%)
					*N* = 271	*N* = 78
Changes in sexual intimacy	563	254 (45%)	84 (33%)	83 (33%)	64 (25%)	29 (46%)
					*N* = 251	*N* = 63
Changes in relationships with friends or coworkers	567	226 (40%)	43 (19%)	82 (36%)	43 (19%)	18 (42%)
					*N* = 224	*N* = 43

* Note: Those who did not answer relevant questions were excluded from the data set. *N* refers to the denominator used for a concern.

**Table 4:** Practical concerns prevalent and ease of accessing help in AYA sample.

Practical Concerns	Number of Respondents Who Answered the Question	Number of Respondents Indicating a Concern about a Practical Challenge (Mild, Moderate, or Big)	Number of Respondents Experiencing a Practical Challenge Whose Concern was ‘Big’	Number of Respondents Experiencing a Practical Challenge Whose Concern was ‘Moderate’	Number of those Experiencing a Practical Challenge Who Sought Help^*^	Number of those Who Sought Help for their Concern that Experienced Some Level of Difficulty (Hard or very Hard to find Help/no Help Obtained) ^*^
Returning to work or school now or in the future	564	351 (62%)	169 (48%)	104 (30%)	136 (39%)	53 (39%)
					*N* = 350	*N* = 136
Difficulty getting health or life insurance	562	229 (41%)	89 (39%)	71 (31%)	65 (29%)	41 (63%)
					*N* = 227	*N* = 65
Getting to and from appointments	563	207 (37%)	33 (16%)	74 (36%)	69 (34%)	13 (19%)
					*N* = 205	*N* = 69
Paying health care bills (e.g., treatment, services, transportation to appointments)	564	180 (32%)	61 (34%)	55 (31%)	76 (43%)	40 (53%)
					*N* = 177	*N* = 75
Taking care of children, elders, or other family members	561	114 (20%)	36 (32%)	28 (25%)	35 (32%)	16 (46%)
					*N* = 109	*N* = 35

* Note: Those who did not answer relevant questions were excluded from the data set. N refers to the denominator used for a concern.

## References

[1] Nass S.J., Beaupin L.K., Demark-Wahnefried W., Fasciano K., Ganz P.A., Hayes-Lattin B., Hudson M.M., Nevidjon B., Oeffinger K.C., Rechis R. (2015). Identifying and addressing the needs of adolescents and young adults with cancer: Summary of an Institute of Medicine workshop. Oncologist.

[2] Statistics Canada (2019). Canadian Vital Statistics, Death Database and Population Estimates.

[3] National Cancer Institute, Adolescent and Young Adult Oncology Progress Review Group (2006). Closing the Gap: Research and Care Imperatives for Adolescents and Young Adults with Cancer.

[4] Keegan T.H.M., Ries L.A., Barr R., Geiger A.M., Dahlke D.V., Pollock B.H., Bleyer W.A., ChB R.D.B.M., Bleyer A., National Cancer Institute Next Steps for Adolescent and Young Adult Oncology Epidemiology Working Group (2016). Comparison of cancer survival trends in the United States of adolescents and young adults with those in children and older adults. Cancer.

[5] Ferrari A., Barr R. (2017). International evolution in aya oncology: Current status and future expectations. Pediatr. Blood Cancer.

[6] De P., Ellison L.F., Barr R.D., Semenciw R., Marrett L.D., Weir H.K., Dryer D., Grunfeld E. (2011). Canadian adolescents and young adults with cancer: Opportunity to improve coordination and level of care. Can. Med. Assoc. J..

[7] Kinahan K., Sanford S., Sadak K., Salsman J., Danner-Koptik K., Didwania A. (2015). Models of Cancer Survivorship Care for Adolescents and Young Adults. Semin. Oncol. Nurs..

[8] Barr R., Ferrari A., Ries L., Whelan J., Bleyer W. (2016). Cancer in Adolescents and Young Adults: A Narrative Review of the Current Status and a View of the Future. JAMA Pediatr..

[9] Bellizzi K.M., Smith A., Schmidt S., Keegan T.H., Zebrack B., Lynch C., Deapen D., Shnorhavorian M., Tompkins B.J., Simon M. (2012). Positive and Negative Psychosocial Impact of Being Diagnosed With Cancer as an Adolescent or Young Adult. Cancer.

[10] Smith A.W., Keegan T., Hamilton A.S., Lynch C., Wu X.-C., Schwartz S., Kato I., Cress R., Harlan L., AYA HOPE Study Collaborative Group (2019). Understanding care and outcomes in adolescents and young adult with Cancer: A review of the AYA HOPE study. Pediatr. Blood Cancer.

[11] Barnett M., A McDonnell G., DeRosa A.P., Schuler T.A., Philip E.J., Peterson L., Touza K.K., Jhanwar S., Atkinson T.M., Ford J.S. (2016). Psychosocial outcomes and interventions among cancer survivors diagnosed during adolescence and young adulthood (AYA): A systematic review. J Cancer Surviv..

[12] Tsangaris E., Johnson J., Taylor R.M., Fern L.A., Bryant-Lukosius D., Barr R., Fraser G., Klassen A. (2014). Identifying the supportive care needs of adolescent and young adult survivors of cancer: A qualitative analysis and systematic literature review. Support Care Cancer.

[13] Fardell J., Patterson P., Wakefield C.E., Signorelli C., Cohn R.J., Anazodo A., Zebrack B., Sansom-Daly U.M. (2018). A Narrative Review of Models of Care for Adolescents and Young Adults with Cancer: Barriers and Recommendations. J. Adolesc. Young Adult Oncol..

[14] Nathan P., Hayes-Lattin B., Sisler J., Hudson M. (2011). Critical issues in transition and survivorship for adolescents and young adults with cancers. Cancer.

[15] Robison L., Hudson M. (2014). Survivors of childhood and adolescent cancer: Life-long risks and responsibilities. Nature Reviews. Cancer.

[16] Tai E., Buchanan N., Townsend J., Fairley T., Moore A., Richardson L.C. (2012). Health status of adolescent and young adult cancer survivors. Cancer.

[17] Mao J., Armstrong K., Bowman M., Xie S., Kadakia R., Farrar J. (2007). Symptom burden among cancer survivors: Impact of age and comorbidity. J. Am. Board Fam. Med..

[18] Quinn G., Gonçalves V., Sehovic I., Bowman M.L., Reed D.R. (2015). Quality of life in adolescent and young adult cancer patients: A systematic review of the literature. Patient Relat. Outcome Meas..

[19] Gupta A.A., Papadakos J., Jones J.M., Amin L., Chang E., Korenblum C., Mina D.S., McCabe L., Mitchell L., Giuliani M. (2016). Reimagining care for adolescent and young adult cancer programs: Moving with the times. Cancer.

[20] Coccia P.F., Altman J., Bhatia S., Borinstein S., Flynn J., George S., Goldsby R.E., Hayashi R., Huang M.S., Johnson R.H. (2012). Adolescent and young adult oncology: Clinical practice guidelines in oncology. J. Natl. Compr. Cancer Netw..

[21] Oeffinger K.C., Mertens A.C., Sklar C.A., Kawashima T., Hudson M.M., Meadows A.T., Friedman D.L., Marina N., Hobbie W., Kadan-Lottick N. (2006). Chronic health conditions in adult survivors of childhood cancer. N. Engl. J. Med..

[22] Woodward E., Jessop M., Glaser A., Stark D. (2011). Late effects in survivors of teenage and young adult cancer: Does age matter?. Ann. Oncol..

[23] Patternson P., McDonald F., Zebrack B., Medlow S. (2015). Emerging Issues Among Adolescent and Young Adult Cancer Survivors. Semin. Oncol. Nurs..

[24] Wilkins K., D’Agostino N., Penney A., Barr R., Nathan P. (2014). Supporting adolescents and young adults with cancer through transitions: Position statement from the Canadian Task Force on Adolescents and Young Adults with Cancer. J. Pediatr. Hematol. Oncol..

[25] Rogers P.C., De Pauw S., Schacter B., Barr R.D. (2013). A Process for Change in the Care of Adolescents and Young Adults with Cancer in Canada. “Moving to Action”: The Second Canadian International Workshop. International Perspectives on AYAO, Part 1. J. Adolesc. Young Adult Oncol..

[26] Barr R., Rogers P., Schacter B. (2011). Adolescents and young adults with cancer: Towards better outcomes in Canada. Cancer.

[27] Keegan T.H.M., Lichtensztajn D.Y., Kato I., Kent E.E., Wu X.-C., West M.M., Hamilton A.S., Zebrack B., Bellizzi K.M., Smith A.W. (2012). Unmet adolescent and young adult cancer survivors information and service needs: A population-based cancer registry study. J. Cancer Surviv..

[28] Zebrack B., Corbett V., Embry L., Aguilar C., Meeske K.A., Block R., Zeman D.T., Cole S., Hayes-Lattin B. (2014). Psychological distress and unsatisfied need for psychosocial support in adolescent and young adult cancer patients during the first year following diagnosis. Psychooncology.

[29] DeRouen M., Smith A.W., Tao L., Bellizzi K.M., Lynch C., Parsons H.M., Kent E.E., Keegan T.H.M., AYA HOPE Study Collaborative Group (2015). Cancer-related information needs and cancer’s impact on control over life influence health-related quality of life among adolescents and young adults with cancer. Psychooncology.

[30] Smith A.W., Parsons H.M., Kent E.E., Bellizzi K., Zebrack B.J., Keel G., Lynch C.F., Rubenstein M.B., Keegan T.H.M. (2013). Unmet support service needs and health-related quality of life among adolescents and young adults with cancer: The AYA HOPE Study. Front. Oncol..

[31] Fitch M., Zomer S., Lockwood G., Louzado C., Moxam R.S., Rahal R., Green E. (2018). Experiences of adult cancer survivors in transitions. Support Care Cancer.

[32] Phillips-Salimi C., Andrykowski M. (2013). Physical and mental health status of female adolescent/young adult survivors of breast and gynecological cancer: A national, population-based, case-control study. Support Care Cancer.

[33] Olsson M., Steineck G., Enskär K., Wilderäng U., Jarfelt M. (2018). Sexual function in adolescent and young adult cancer survivors—A population-based study. J Cancer Surviv..

[34] Salsman J., Garcia S., Yanez B., Sanford S., Snyder M., Victorson D. (2014). Physical, emotional, and social health differences between posttreatment young adults with cancer and matched healthy controls. Cancer.

[35] Nowe E., Stöbel-Richter Y., Sender A., Leuteritz K., Friedrich M., Geue K. (2017). Cancer-related fatigue in adolescents and young adults: A systematic review of the literature. Crit. Rev. Oncol. Hematol..

[36] Hauken M., Holsen I., Fismen E., Larsen T. (2015). Working toward a good life as a cancer survivor: A longitudinal study on positive health outcomes of a rehabilitation program for young adult cancer survivors. Cancer Nurs..

[37] Smith A.W., Bellizzi K.M., Keegan T.H., Zebrack B., Chen V.W., Neale A.V., Hamilton A.S., Shnorhavorian M., Lynch C.F. (2013). Health-related quality of life of adolescent and young adult patients with cancer in the United States: The adolescent and young adult health outcomes and patient experience study. J. Clin. Oncol..

[38] Wettergren L., Kent E.E., Mitchell S.A., Zebrack B., Lynch C., Rubenstein M.B., Keegan T.H.M., Wu X.-C., Parsons H.M., Smith A.W. (2017). Cancer negatively impacts on sexual function in adolescents and young adults: The AYA HOPE study. Psychooncology.

[39] Carter J., Lacchetti C., Andersen B.L., Barton D.L., Bolte S., Damast S., Diefenbach M.A., Duhamel K., Florendo J., A Ganz P. (2018). Interventions to address sexual problems in people with cancer: American Society of Clinical Oncology clinical practice guideline adaptation of Cancer Care Ontario guideline. J. Clin. Oncol..

[40] Barbera L., Zwaal C., Elterman D., McPherson K., Wolfman W., Katz A., Matthew A. (2017). Interventions to address sexual problems in people with cancer. Curr. Oncol..

[41] Nathan P.C., Dilley K., Harvey J., Jacobsen C., McKinley K., Millham A.K., Moore I., Okcu M.F., Brouwers P., Armstrong F.D. (2007). Long-term Follow-up Guidelines Task Force on Neurocognitive/Behavioral Complications After Childhood Cancer. Guidelines for identification of, advocacy for, and intervention in neurocognitive problems in survivors of childhood cancer: A report from the Children’s Oncology Group. Arch. Pediatr. Adolesc. Med..

[42] Ahles T.A., Root J.C., Ryan E.L. (2012). Cancer-and cancer treatment-associated cognitive change: An update on the state of the science. J. Clin. Oncol..

[43] Klaver K.M., Duijts S.F.A., Engelhardt E.G., Geusgens C.A.V., Aarts M.J.B., Ponds R.W.H.M., Van Der Beek A.J., Schagen S.B. (2019). Cancer-related cognitive problems at work: Experiences of survivors and professionals. J. Cancer Surviv..

[44] Treanor C., McMenamin Ú.C., O’Neill R.F., Cardwell C.R., Clarke M.J., Cantwell M., Donnelly M. (2016). Non-pharmacological interventions for cognitive impairment due to systemic cancer treatment. Cochrane Database Syst. Rev..

[45] Linden W., Vodermaier A., Mackenzie R., Greig D. (2012). Anxiety and depression after cancer diagnosis: Prevalence rates by cancer type, gender, and age. J. Affect. Disord..

[46] Lang M., David V., Giese-Davis J. (2015). The age conundrum: A scoping review of younger age or adolescent and young adult as a risk factor for clinical distress, depression, or anxiety in cancer. J. Adolesc. Young Adult Oncol..

[47] Mor V., Allen S., Malin M. (1994). The psychosocial impact of cancer on older versus younger patients and their families. Cancer.

[48] Zebrack B., Isaacson S. (2012). Psychosocial Care of Adolescent and Young Adult Patients With Cancer and Survivors. J. Clin. Oncol..

[49] Hauken M., Larsen T., Holsen I. (2013). Meeting reality: Young adult cancer survivors’ experiences of reentering everyday life after cancer treatment. Cancer Nurs..

[50] Geue R.M.D.-P.K., Sender A., Schmidt R., Richter D., Hinz A., Schulte T., Brähler E., Stöbel-Richter Y. (2014). Gender-specific quality of life after cancer in young adulthood: A comparison with the general population. Qual. Life Res..

[51] Bolte S., Zebrack B. (2008). Sexual Issues in Special Populations: Adolescents and Young Adults. Semin. Oncol. Nurs..

[52] Geue K., Schmidt R., Sender A., Sauter S., Friedrich M. (2015). Sexuality and romantic relationships in young adult cancer survivors: Satisfaction and supportive care needs. Psycho-Oncol..

[53] Carpentier M., Fortenberry J. (2010). Romantic and Sexual Relationships, Body Image, and Fertility in Adolescent and Young Adult Testicular Cancer Survivors: A Review of the Literature. Journal of Adolescent Health. **2010**, *47*, 115–125. J. Adolesc. Health.

[54] Brierley M., Sansom-Daly U., Baenziger J., McGill B., Wakefield C. (2019). Impact of physical appearance changes reported by adolescent and young adult cancer survivors: A qualitative analysis. Eur. J. Cancer Care.

[55] Evan E.E., Zeltzer L.K. (2006). Psychosocial dimensions of cancer in adolescents and young adults. Cancer.

[56] Richter D., Koehler M., Friedrich M., Hilgendorf I., Mehnert A., Weissflog G. (2015). Psychosocial interventions for adolescents and young adult cancer patients: A systematic review and meta-analysis. Crit. Rev. Oncol. Hematol..

[57] Martins A., Taylor R., Lobel B., McCan B., Soanes L., Whelan J., Fern L. (2019). P53 Sex, body image and relationships: Young people with cancer information and support preferences. BMJ Paediatr. Open.

[58] Mairghread C., Lewin J., Lazarakis S., Thompson K. (2019). Overlooked Minorities: The Intersection of Cancer in Lesbian, Gay, Bisexual, Transgender, and/or Intersex Adolescents and Young Adults. J. Adolesc. Young Adult Oncol..

[59] Parsons H.M., Harlan L.C., Lynch C.F., Hamilton A.S., Wu X.-C., Kato I., Schwartz S., Smith A.W., Keel G., Keegan T.H. (2012). Impact of Cancer on Work and Education Among Adolescent and Young Adult Cancer Survivors. J. Clin. Oncol..

[60] Mentschke L., Leuteritz K., Daneck L., Breuer N., Sender A., Friedrich M., Nowe E., Stöbel-Richter Y., Geue K. (2017). Cancer and Career? A Qualitative Study of Job Status of Young Adult Cancer Survivors. Psychother. Psychosom. Med. Psychol..

[61] Ketterl T., Syrjala K.L., Casillas J., Jacobs L.A., Palmer S.C., McCabe M.S., Ganz P.A., Overholser L., Partridge A.H., Rajotte E.J. (2019). Lasting effects of cancer and its treatment on employment and finances in adolescent and young adult cancer survivors. Cancer.

[62] Kosola S., McCarthy M., McNeil R., Orme L., Drew S., Sawyer S. (2018). Early Education and Employment Outcomes after Cancer in Adolescents and Young Adults. J. Adolesc. Young Adult Oncol..

[63] Osborn M.P., Johnson R., Thompson K., Anazodo A., Albritton K., Ferrari A., Stark D. (2019). Models of Care for Adolescent and Young Adult Cancer Programs. Pediatr. Blood Cancer.

[64] Coccia P.F., Pappo A.S., Beaupin L., Borges V.F., Borinstein S., Chugh R., Dinner S., Folbrecht J., Frazier A.L., Goldsby R. (2018). Adolescent and Young Adult Oncology, Version 2.2018, NCCN Clinical Practice Guidelines in Oncology. J. Natl. Compr. Cancer Netw..

[65] Ferrari A., Thomas D.M., Franklin A.R., Hayes-Lattin B.M., Mascarin M., Van Der Graaf W., Albritton K.H. (2010). Starting an adolescent and young adult program: Some success stories and some obstacles to overcome. J. Clin. Oncol..

[66] Reed D.R., Block R.G., Johnson R. (2014). Creating an adolescent and young adult cancer program: Lessons learned from pediatric and adult oncology practice bases. J. Natl. Compr. Cancer Netw..

